# Implementation of internet-delivered cognitive behaviour therapy for pediatric obsessive-compulsive disorder: Lessons from clinics in Sweden, United Kingdom and Australia

**DOI:** 10.1016/j.invent.2020.100308

**Published:** 2020-01-27

**Authors:** Kristina Aspvall, Fabian Lenhard, Karin Melin, Georgina Krebs, Lisa Norlin, Kristina Näsström, Amita Jassi, Cynthia Turner, Elizabeth Knoetze, Eva Serlachius, Erik Andersson, David Mataix-Cols

**Affiliations:** a Centre for Psychiatry Research, Department of Clinical Neuroscience, Karolinska Institutet, & Stockholm Health Care Services, Region Stockholm, Sweden; b Institute of Neuroscience and Physiology, The Sahlgrenska Academy, University of Gothenburg, Gothenburg, Sweden; c Department of Child and Adolescent Psychiatry, CAP Specialized unit, Sahlgrenska University Hospital, Gothenburg, Sweden; d Institute of Psychiatry, Psychology & Neuroscience, MRC Social, Genetic and Developmental Psychiatry Centre King's College, London, United Kingdom; e National Specialist OCD, BDD and related disorders clinic, Maudsley Hospital, London, United Kingdom; f Primary Care Clinical Unit, Faculty of Medicine, The University of Queensland, Brisbane, Australia; g School of Psychology, The University of Queensland, Brisbane, Australia; h Division of Psychology, Department of Clinical Neuroscience, Karolinska Institutet, Stockholm, Sweden

**Keywords:** Obsessive-compulsive disorder, Cognitive behaviour therapy, Exposure with response prevention, Internet, Self-help, Child, Adolescent

## Abstract

Obsessive-compulsive disorder (OCD) can be successfully treated with cognitive behaviour therapy (CBT). However, as few patients have access to CBT, there is a strong push to develop and evaluate scalable and cost-effective internet-delivered interventions. BIP OCD is a therapist-guided online CBT intervention for pediatric OCD that has shown promise in trials conducted at a single site in Stockholm, Sweden. In this study, we evaluated if BIP OCD is an acceptable, feasible, and effective treatment in other countries and clinical contexts. Thirty-one patients were recruited at three different sites; a specialist OCD clinic in Gothenburg (Sweden), a specialist OCD clinic in London (United Kingdom), and a university-based clinic in Brisbane (Australia). Acceptability and feasibility measures included treatment adherence and feedback from therapists. Clinician assessments were conducted at baseline, post-treatment, and 3-month follow-up. The average module completion for the participants was 8.1/12 (SD = 3.2) and the majority of patients completed the BIP OCD treatment (100% in Gothenburg, and 55.6% in both London and Brisbane). Pooling data from the three sites, the within-group effect sizes from baseline to post-treatment on the Children's Yale-Brown Obsessive-Compulsive Scale were in the expected range (bootstrapped Cohen's *d* = 1.78; 95% CI 1.18–2.39), with an additional symptom reduction to the 3-month follow-up (bootstrapped Cohen's *d* = 0.27; 95% CI 0.02–0.51). Participating therapists identified both advantages and difficulties supporting patients in this digital format. The results of this study suggest that the treatment effects obtained in the original BIP OCD trials can be generalized to other clinical contexts nationally and internationally. Lessons learned provide important information for successful implementation of BIP OCD in regular healthcare contexts.

## Highlights

• ICBT for pediatric OCD was evaluated within three different clinical contexts.
• The sample primarily consisted of clinician-referred patients.
• The majority of patients completed the treatment and had clinical meaningful effects.
• Therapists identified advantages and difficulties supporting patients online.
• Lessons learned provide important information for successful implementation.

## 1 Introduction

Obsessive-compulsive disorder (OCD) is a psychiatric disorder characterized by distressing and time-consuming obsessions and compulsions (American Psychiatric Association, 2013). Obsessions are unwanted recurrent and intrusive thoughts, images or impulses (eg. fear of contamination or harming important people) and compulsions are repetitive behaviours that aim to reduce or neutralize the distress caused by the obsessions (eg. excessive washing, checking or mental rituals). In children and adolescents, OCD is associated with functional impairments in many important areas, such as education (Pérez-Vigil et al., 2018), at home and in social relationships (Valderhaug and Ivarsson, 2005). Since about 70% of individuals have a symptom onset during childhood (Taylor, 2011), and OCD can be chronic if left untreated (Pinto et al., 2006), there is broad consensus in the field that early interventions should be prioritized (Fineberg et al., 2019).

There is solid evidence that cognitive behaviour therapy (CBT) is an effective psychological treatment for children and adolescents with OCD, with large effect sizes compared to both waitlist and active control conditions (*g* = 0.93–1.53; Öst et al., 2016). However, despite expert consensus and guidelines recommending CBT as a first-line treatment for the disorder (Geller and March, 2012; National Institute for Health and Care Excellence, 2005; The National Board of Health and Welfare, 2017), accessibility is low and a majority of OCD sufferers do not have access to this treatment (Krebs et al., 2015; Nair et al., 2015; Schwartz et al., 2013). Possible barriers include the shortage of trained CBT therapists, high costs of treatment, and geographical factors (Marques et al., 2010; Shapiro et al., 2003).

In an attempt to increase access to effective treatment, several options to deliver CBT online have been developed (Babiano-Espinosa et al., 2019; Wootton, 2016). One of these approaches is therapist-guided Internet-delivered CBT (ICBT) which has been evaluated for adults in Australia (Wootton et al., 2013), and Sweden (Andersson et al., 2012; Andersson et al., 2015). For children and adolescents with OCD, only one therapist-guided ICBT intervention has been developed and evaluated to date, called BIP OCD, which was developed in Stockholm, Sweden (Aspvall et al., 2018; Lenhard et al., 2017a; Lenhard et al., 2014). BIP OCD has been evaluated in two open feasibility studies with promising results for both children (*N* = 11, within-group Cohen's *d* = 1.86; Aspvall et al., 2018) and adolescents (*N* = 21, within-group Cohen's *d* = 2.29; Lenhard et al., 2014), and showed superiority and cost-effectiveness compared to waitlist in a randomized controlled trial (RCT; *N* = 67, between-group Cohen's *d* = 0.69; Lenhard et al., 2017a; Lenhard et al., 2017b). A further RCT is currently ongoing, aiming to establish if BIP OCD in a stepped care approach is as efficacious (i.e. non-inferior) and cost-effective compared to standard face-to-face CBT for young people with OCD (Aspvall et al., 2019).

BIP OCD has so far only been evaluated in a Swedish clinical-academic setting where the intervention was developed, with the majority of participants being self-referred. There is however a need move from efficacy trials conducted in academic settings to effectiveness trials, where the treatments are evaluated in regular healthcare settings. This may be especially important in the case of ICBT as some research has suggested that the effects are smaller when delivered in routine care compared to research trials (Romijn et al., 2019). Effectiveness studies of ICBT have been done both for adults with several mental health conditions (Andersson and Hedman, 2013; Titov et al., 2018), and for children with anxiety disorders (Jolstedt et al., 2018a; Jolstedt et al., 2018b; Moor et al., 2019), but comparable studies are lacking in pediatric OCD. Thus, an important next step is to investigate if the promising effects of BIP OCD are generalizable to other countries and different healthcare contexts, and more diverse patient groups. The aim of this study was therefore to evaluate the acceptability, feasibility, and preliminary effectiveness of implementing BIP OCD in three different clinics in Sweden, United Kingdom and Australia, where the intervention had not been tested before, and where patients were mainly clinician-referred. Specifically, we report on treatment adherence, outcome data, and therapists' views on the advantages and disadvantages of this treatment modality.

## 2 Methods

### 2.1 Study design

BIP OCD was evaluated at three different sites using an open trial design (repeated measurements). As per the original BIP OCD trials, the duration of the treatment was 12 weeks. The sites were located in Gothenburg (specialized Child and Adolescent Psychiatry unit for OCD at Sahlgrenska University Hospital, Sweden), London (National Specialist Clinic for Young People with OCD, BDD and related disorders at South London and Maudsley NHS Foundation Trust, United Kingdom), and Brisbane (the School of Psychology at University of Queensland, Australia). The clinic in Gothenburg is a specialized unit that receives both self-referrals and clinician-referrals from Child and Adolescent Mental Health Services within the region, but also accepts referrals from other parts of Sweden. The London clinic is a national service clinic that receives referrals from all parts of United Kingdom, and tend to see severe or complex cases where a majority of the patients have had previous treatment for OCD. The Brisbane site is a University clinic, that tends to attract patients who live out of regional cities where there is limited access to healthcare services, and receives mainly self-referrals from parents who feel that their children would be unwilling to go to a clinic and instead engage better with an online intervention. Each site received local ethical approval (Gothenburg ID 2016/2161-32; London ID 16/WA/0064; Brisbane ID UQ_IHREA_2015000406).

### 2.2 Participants

Inclusion and exclusion criteria were similar across sites, with small differences, described here. Inclusion criteria were a primary diagnosis of OCD as defined by DSM-5 (Gothenburg and Brisbane; American Psychiatric Association, 2013) or ICD-10 (London; World Health Organization, 1992), and a total score of ≥16 on the Children's Yale-Brown Obsessive-Compulsive Scale (CY-BOCS; Scahill et al., 1997). The age criteria were between 7 and 17 years (Gothenburg) or 12 and 18 years (London and Brisbane). Additional criteria were ability to read and write Swedish (Gothenburg) or English (London and Brisbane), daily access to the Internet, a parent or caregiver that could participate in the treatment, a requirement to live in the country where the trial was conducted, and for participants on psychotropic medication, a stable dose for the last six weeks prior to baseline assessment.

Exclusion criteria were a comorbid diagnosis of global learning disabilities, autism spectrum disorder, psychosis or bipolar disorder, anorexia nervosa, or a severe neurological condition; active suicidal ideation; ongoing substance dependence (London and Brisbane); not being able to read or understand the basics of the ICBT material; received CBT for OCD within the last year (Gothenburg); presenting with very severe OCD symptoms (a total score of >35 on the CY-BOCS, Brisbane); and ongoing psychological treatment for OCD or an anxiety disorder. All participants gave written informed consent to participate prior to inclusion.

### 2.3 Measures

#### 2.3.1 Diagnostic measures

The diagnostic interview was done using the Mini International Neuropsychiatric Interview for Children and Adolescents (MINI-KID; Sheehan et al., 2010) in Gothenburg and Brisbane, and the Anxiety Disorders Interview Schedule for the DSM-IV (ADIS; Albano and Silverman, 1996) in London.

#### 2.3.2 Treatment adherence and therapist support time

The number of completed modules and the number of individuals classed as treatment completers were used as measures of treatment adherence. Treatment completers were defined as individuals completing the first active treatment module, containing exposure with response prevention (ERP).

The amount of therapist time supporting each family was recorded. It includes time spent on correspondence with both child and the parents as well as additional phone calls if required. Both the average therapist time per family for the whole treatment and the average therapist time per week in treatment (therapist time divided by number of modules completed) were calculated.

#### 2.3.3 Primary outcome measure

The primary outcome measure was the CY-BOCS (Scahill et al., 1997), which is a semi-structured, clinician-rated interview to determine the severity of OCD symptoms in children and adolescents. It consists of a symptom checklist and a 10-item severity scale. Each item is rated on a 5-point scale, which yields a total severity score ranging from 0 to 40, with higher scores indicating more severe symptoms. It has good to excellent interrater reliability, high internal consistency and good short-term test-retest reliability (Cook et al., 2015; Scahill et al., 1997; Storch et al., 2004).

#### 2.3.4 Secondary outcome measures

The clinician-rated CGAS (Shaffer et al., 1983) is a clinician-rated global measure of functional impairment. It is scored from 0 to 100, with higher scores indicating better functioning. CGAS is reliable between raters and across time, and has demonstrated both discriminant and concurrent validity (Shaffer et al., 1983).

The Children's Obsessional Compulsive Inventory Revised – Parent (ChOCI-R-P; Uher et al., 2008), the revised version of the ChOCI (Shafran et al., 2003), is a 32-item parent-rated measure of OCD symptom severity that includes both symptoms and severity items. The total impairment score is based on the 12 severity items and range from 0 to 48, with higher scores indicating more OCD symptoms. ChOCI-R-P has demonstrated good internal consistency and criterion validity (Uher et al., 2008).

The Work and Social Adjustment Scale – Youth and Parent versions (WSAS-Y/P; Jassi et al., in press) is a 5-item measure of general functional impairment related to psychopathology. The WAS-Y/P is a child and parent adaptation of the adult WSAS measure (David Mataix-Cols et al., 2005; Mundt et al., 2002). The total score ranges from 0 to 40. Both the child and parent versions possess good internal consistency, test-retest reliability, convergent validity, and sensitivity to change (Jassi et al., in press).

#### 2.3.5 Feedback from therapists

The therapists provided feedback about the feasibility and acceptability of delivering BIP OCD within their services by answering open questions about advantages and/or concerns they had with the treatment and what they considered important in implementing the treatment (see supplement S3). The feedback from therapists was summarized and grouped into specific themes.

### 2.4 Procedures

Participants were consecutively recruited from routine clinical referrals to the Gothenburg and London clinics. In Brisbane, participants could either be referred by a clinician or be self-referred, and the trial was advertised both online, in media, and via flyers in offices of general practitioners. Eligible participants were assessed by a clinician either at a face-to-face appointment (Gothenburg and London) or via the phone (Brisbane). The assessment included a diagnostic interview using the MINI-KID (Sheehan et al., 2010) or the ADIS (Albano and Silverman, 1996), the clinician-rated measures CY-BOCS for baseline OCD symptom severity and CGAS (Shaffer et al., 1983) for global functioning, and all self- and parent-rated measures. All participants and parents/caregivers provided written informed consent to participate prior to inclusion during this visit.

**Fig. 1:**
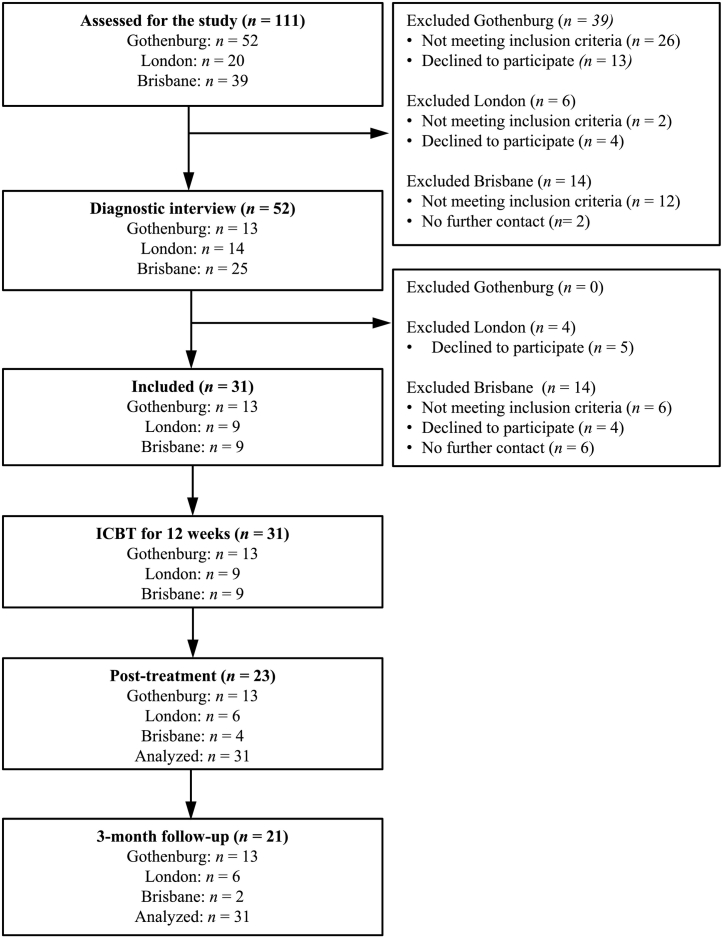
Study flow chart.

After 12 weeks of treatment, a post-treatment assessment was conducted including all clinician-, self- and parent-rated measures. The same procedure was repeated 12 weeks later (3-month follow-up). The assessments at post-treatment and 3-month follow-up were conducted by a different clinician than the treating therapist. Fig. 1 shows the study flow with an overview of the inclusion and assessment points, by study site.

### 2.5 Intervention

Two different language versions of BIP OCD exist. The London and Brisbane sites employed the English version of BIP OCD, which is a translation of the original BIP OCD intervention directed to adolescents by Lenhard et al. (2017a). This version of BIP OCD consisted of 12 modules for the adolescent and five parallel modules for parents. The Gothenburg site employed an updated version of BIP OCD, which was expanded to suit both children and adolescents with OCD, based on pilot work by Aspvall et al. (2018). The updated BIP OCD version, only available in Swedish, included more parent modules (12 modules) and introduces the core treatment ingredient ERP earlier. See Table 1 for an overview of the treatment content.

**Table 1:** Treatment overview.

Chapter	Swedish BIP OCD		English BIP OCD	
	Child/Adolescent	Parent	Adolescent	Parent
Education				
1	What is OCD?	Education about OCD and the OCD circle	Introduction to ICBT	Introduction to ICBT
2	How to get rid of OCD?	About CBT and treatment goals	Education about OCD	Education about OCD
3	What is exposure?	About exposure and response prevention	The OCD circle	

Exposure with response prevention				
4	Testing exposure	Parent strategies during exposure	Building an exposure hierarchy	
5	Continue with exposure	More about exposure	About exposure and response prevention	About exposure and response prevention
6	When OCD affects the family	About family accommodation	Testing exposure	Parental strategies during exposure
7	More exposure	Motivation and parent strategies	More exposure	
8	Coping with obsessions	Coping with obsessions	Exposure, frequent problems and solutions	About family accommodation
9	New steps with exposure	Evaluate the treatment	New steps with exposure	
10	Using re-exposure techniques	Using re-exposure techniques	Coping with obsessions	
11	Perform a more difficult exposure	Perform a more difficult exposure	Final exposure exercises	

Relapse prevention				
12	Plan for the future	Plan for the future	Relapse prevention and lessons learned	

Abbreviations: OCD, obsessive-compulsive disorder; CBT, cognitive behaviour therapy; ICBT, internet-delivered cognitive behaviour therapy.

To enable access to BIP OCD, all participants and parents/legal guardians were given separate logins to a secure webpage through which the treatment was delivered. The intervention followed recommendations from established guidelines (Geller and March, 2012; National Institute for Health and Care Excellence, 2005; The National Board of Health and Welfare, 2017), and included psychoeducation, ERP, and relapse prevention. Each module consists of text, brief video clips, animations, examples, and exercises that helped the participant and parents to learn about OCD, create an ERP hierarchy and how to implement and practice ERP tasks in everyday situations. Throughout the treatment, each family had contact with a designated therapist who provided asynchronous, predominantly written, support with occasional telephone calls if needed. Therapists assisted with planning, problem-solving and encouraged compliance with ERP and homework tasks. The following video illustrates the main features of BIP OCD: https://vimeo.com/355965105/b3d5d1c439.

All therapists (three in Gothenburg, two in London and one in Brisbane) were highly experienced in the delivery of face-to-face CBT for young people with OCD within specialized care. The Gothenburg team had a two-day workshop prior to starting ICBT as well as weekly supervision during the trial from two of the authors (KA and FL), while the London and Brisbane teams received training videos about BIP OCD and videoconference supervision on demand.

### 2.6 Data analysis

A series of mixed-effect regression analyses with maximum likelihood estimation were conducted on the outcome measures to detect statistical change between the assessment points (Gueorguieva and Krystal, 2004; Hesser, 2015). Adding site, number of completed chapters or therapist time as a covariate did not improve the model fit, and were not included in the final model. Effect sizes (Cohen's *d*) was estimated with the m_effectsize command in STATA developed by Karolinska Institutet Biostatistics Core Facility and available at www.imm.ki.se/biostatistics/stata. The α value (2-tailed) was set at *p* < .05 for all analyses. A sensitivity analysis was made as recommended when the missing data is above 5% (Jakobsen et al., 2017). This was done using multiple imputation by chained equations with predictive mean matching on 20 imputed datasets, to check if the raw versus imputed datasets yielded similar results. Statistical analyses were conducted using STATA version 14.2 (StataCorp LLC).

## 3 Results

### 3.1 Sample characteristics and study flow

Thirty-one patients participated in the current study (Gothenburg: *n* = 13; London: *n* = 9; Brisbane: *n* = 9). Demographic and clinical characteristics of the patients are presented both for the total group and by study site in Table 2. The average age was 14.0 years (SD = 2.3), 41.9% (*n* = 10) had received previous CBT for OCD, and more than half (54.8%; *n* = 17) had at least one comorbid disorder, out of which specific phobia was the most prevalent. All patients in Gothenburg and London, and 33.3% (*n* = 3) in Brisbane were clinician-referred. The other Brisbane participants were self-referred following advertising of the study. As shown in Fig. 1, Gothenburg had complete primary outcome data on the clinician-rated measures on all assessment points, London had 66.7% complete data (*n* = 6/9) on both post-treatment and 3-month follow-up assessments, and Brisbane had 44.4% complete data (*n* = 4/9) at post-treatment and 22.2% (*n* = 2/9) at the 3-month follow-up assessment.

**Table 2:** Baseline demographic and clinical characteristics of the sample (*N* = 31).

	Total sample	Gothenburg (*n* = 13)	London (*n* = 9)	Brisbane (*n* = 9)
Gender, *N* (%)				
Girls	21 (67.7)	7 (53.9)	6 (66.7)	8 (88.9)
Age (years)				
Mean (SD), range	14.0 (2.3), 8–18	13.2 (2.6), 8–17	15.6 (2.0), 13–18	13.6 (1.2), 12–16
Distance to clinic (km)				
Median, range	16, 2–2435	14, 2–41	13, 2–438	1443, 9-2435^a^
Main contact person, *N* (%)				
Mother	25 (80.7)	8 (61.6)	8 (88.9)	10 (100)
Psychotropic medication for OCD, *N* (%)				
Previous	3 (9.7)	0 (0)	1 (11.1)	2 (22.2)
Current	7 (22.6)	2 (15.4)	5 (55.6)	0 (0)
Previous psychological treatment, *N* (%)				
CBT for OCD	10 (41.9)	3 (23.1)	4 (44.4)	3 (33.3)
CBT for other	4 (12.9)	0 (0)	1 (11.1)	3 (33.3)
Other	4 (12.9)	1 (7.7)	1 (11.1)	2 (22.2)
Presence of comorbid diagnosis, *N* (%)				
Yes	17 (54.8)	5 (38.5)	5 (55.6)	7 (77.8)
Frequency of comorbid diagnosis, N (%)				
BDD	1 (3.2)	0 (0)	1 (11.1)	0 (0)
Skin-picking disorder	1 (3.2)	1 (7.7)	0 (0)	0 (0)
Specific phobia	8 (25.8)	2 (15.4)	0 (0)	6 (66.7)
Social anxiety disorder	5 (16.1)	1 (7.7)	0 (0)	4 (44.4)
Separation anxiety	1 (3.2)	0 (0)	1 (11.1)	0 (0)
GAD	3 (9.7)	1 (7.7)	1 (11.1)	1 (11.1)
Agoraphobia	1 (3.2)	1 (7.7)	0 (0)	0 (0)
Depressive disorder	3 (9.7)	1 (7.7)	2 (22.2)	0 (0)
Tic disorder	2 (6.5)	0 (0)	0 (0)	2 (22.2)
ADHD	2 (6.5)	1 (7.7)	0 (0)	1 (11.1)
CY-BOCS total score				
Mean (SD), range	24.6 (3.5), 17–31	24.1 (3.2), 17–29	24.9 (4.7), 18–30	25.0 (2.9), 21–31
Referral source				
Clinician-referred	25 (80.1)	13 (100)	9 (100)	3 (33.3)^a^

Note: Site differences were calculated with a Bonferroni-corrected analysis. Subscripts (^a^) indicates a significant difference from the other sites.

Abbreviations: ADHD, Attention Deficit/Hyperactivity Disorder; CY-BOCS, Children's Yale-Brown Obsessive-Compulsive Scale.

### 3.2 Treatment adherence and therapist support time

The average module completion was 8.1/12 (SD = 3.2). There was no significant effect of site on module completion (F(2) = 1.22, *p* = .31). Patients completed on average 76% (M = 9.1/12, SD = 2.3) of the modules in Gothenburg, 65% (M = 7.8/1, SD = 3.0) in London, and 58% (M = 7/12, SD = 4.2) in Brisbane. The parents completed an average of 77% (M = 9.2/12, SD 2.5) of the modules in Gothenburg, 62% (M = 3.1/5, SD = 2.1) in London, and 78% (M = 3.9/5, SD 1.3) in Brisbane.

The number of treatment completers differed by site (χ2 (2, *N* = 31) = 8.79, *p* = .02). Gothenburg had a higher degree of treatment completers (100%; *n* = 13) compared with London (55.6%; *n* = 5; *p* < .01) and Brisbane (55.6%; n = 5, *p* = .01). The average therapist support time for the whole treatment were 411.8 min (SD = 178.8, median = 360) in Gothenburg, 159.6 min (SD = 107.2, median = 98.0) in London, and 153.3 min (SD = 117.0, median = 146.0) in Brisbane. This corresponds to an average therapist time per week in treatment of 44.6 min (SD = 11.7, median = 41.5) in Gothenburg, 19.1 min (SD = 7.0, median = 20.3) in London, and 19.3 min (SD = 8.6, median = 19.7) in Brisbane, which is a significant effect of site on total therapist time (F(2) = 25.64, *p* < .000) with Gothenburg spending more time per patient than in London (*p* < .001) and Brisbane (*p* < .001).

### 3.3 Clinical outcome measures

Table 3 shows the observed means and standard deviations on all measures by assessment point, and the estimated mean values and effect sizes between baseline to post-treatment and post-treatment to the 3-month follow-up. For descriptive purposes, the observed values by site are reported in the supplement (S1).

**Table 3:** Observed means and standard deviations, estimated means and effect sizes (bootstrapped Cohen's *d*).

Measure	Observed values						Estimated values		Effect sizes			
	Baseline		Post-treatment		3-month follow-up		Post-treatment	3-month follow-up	Baseline - Post-treatment		Post-treatment - 3-month follow-up	
	Mean (SD)		Mean (SD)		Mean (SD)		Mean	Mean	Cohen's *d* (95% CI)		Cohen's *d* (95% CI)	
CY-BOCS	24.58	(3.54)	15.61	(6.81)	12.95	(8.75)	15.80⁎⁎	15.61⁎	1.78	(1.18, 2.39)	0.27	(0.02, 0.51)
CGAS	52.35	(7.41)	61.14	(10.03)	64.86	(13.97)	61.89⁎⁎	61.29	1.15	(0.76, 1.54)	0.29	(−0.08, 0.65)
ChOCI-R-P	25.72	(8.48)	18.68	(9.08)	15.67	(9.97)	18.14⁎⁎	18.68	0.92	(0.42, 1.43)	0.23	(−0.05, 0.50)
WSAS-Y	13.37	(6.49)	9.05	(5.70)	7.70	(5.90)	8.61⁎⁎	9.11	0.77	(0.27, 1.27)	0.30	(−0.18, 0.77)
WSAS-P	12.55	(8.94)	9.10	(8.00)	8.44	(7.54)	7.86⁎⁎	8.99	0.56	(0.27, 0.84)	0.08	(−0.29, 0.45)

Abbreviations: CY-BOCS, Children Yale-Brown Obsessive-Compulsive Scale; CGAS, Children's Global Assessment Scale; ChOCI-R-P, Children's Obsessional Compulsive Inventory – Revised – Parent version; WSAS-Y, Work, and Social Adjustment Scale – Youth version, WSAS-P, Work, and Social Adjustment Scale – Parent version.

⁎ *p* < .01.

⁎⁎ *p* < .001.

The analyses showed significant improvements on all the outcome measures between baseline and post-treatment, with a large within-group effect size on the CY-BOCS (bootstrapped Cohen's *d* = 1.78; 95% CI 1.18–2.39) and the secondary outcome measures, except on WSAS-P, which showed a moderate effect size. All results were sustained at the 3-month follow-up, and an additional reduction from post-treatment to the 3-month follow-up on the CY-BOCS (bootstrapped Cohen's *d* = 0.27; 95% CI 0.02–0.51). The results from the sensitivity analysis based on the multiple imputed datasets yielded similar results, except for on WSAS-P which was not significant from baseline to post-treatment. Details from the sensitivity analysis are presented in the supplement (S2).

### 3.4 Feedback from the therapists

Among the advantages reported by the therapists were the efficient use of therapist time, convenience and availability for families, and that the parents are included as co-therapists. Reported difficulties were to get a detailed assessment of how much some patients adhered to the ERP exercises, and to sufficiently engage and influence the patients through the written online format compared with the traditional face-to-face format. Therapists perceived that the treatment was less suitable for patients with more complex symptoms, such as mental compulsions, rigid rules, and reading/writing compulsions, as well as for those who have not responded sufficiently to previous treatment or were ambivalent to CBT. Important aspects that were identified for successful implementation of BIP OCD in routine healthcare were to develop guidelines regarding which patients may be best suited to ICBT in terms of patient motivation prior to initiating ICBT, have routines for risk monitoring, and the importance of therapist training on how to deliver ICBT (e.g. how to give optimal support online and cope with inactive participants). Detailed therapist feedback by study site is presented in the supplement (S3).

## 4 Discussion

The aim of this study was to extend findings from the previously conducted BIP OCD trials to other countries and clinics within different healthcare contexts. Prior to this study, BIP OCD had only been evaluated in a clinical-academic setting in Stockholm, where it was originally developed. In contrast to previous BIP OCD trials, which mainly recruited self-referred participants, a defining feature of the study was the enrolment of primarily clinician-referred patients. The current study therefore fills an important clinical research gap by moving beyond efficacy trials and beginning to investigate effectiveness of the intervention in regular healthcare contexts.

Overall, we observed a high degree of adherence to the BIP OCD intervention with at least 62–78% of treatment modules completed, and a majority of patients being classified as completers (56–100%). The Gothenburg site had higher degree of treatment completers than London and Brisbane. This was partly expected as the updated version of BIP OCD used in Gothenburg introduces the active treatment component ERP earlier within the intervention (in module 4 versus module 6), which may have contributed to greater treatment adherence in this site. The higher level of parental involvement (12 versus 5 modules) could also be an important aspect, but the importance of parental involvement in ICBT for pediatric OCD is still an empirical question. The differences in versions of BIP OCD and adherence between the sites are limitations of the study. Another significant difference was that the therapists in Gothenburg spent substantially more time on each family (45 versus 19 min per patient per week). This may reflect the greater number of parental modules in the updated version of BIP OCD. An additional difference was that the clinicians in the Gothenburg site received an initial two-day workshop on how to deliver BIP OCD as well as weekly supervision during the trial, which was more systematic and frequent than in the other two sites. Altogether, this may have contributed to the higher degree of treatment completers and lack of data loss at this particular site. We recommend that future studies use the most recent version of BIP OCD, that clinicians supporting the families receive specific training before starting treatment, and that they have several patients to be efficient in the online delivery. Furthermore, regular supervision of clinicians is recommended, just as in regular clinic-based CBT.

Some of the observed site differences in treatment completers and data loss might be attributable, at least in part, to the different healthcare systems. Although Sweden, the United Kingdom, and Australia all have tax-funded universal healthcare systems, each have a different financial model. The healthcare in Sweden is mostly provided by the tax-funded county councils. The specialist OCD clinic in Gothenburg receives referrals from primary care and from local child and adolescent mental health units (Melin et al., 2015), and typically sees both treatment naïve and treatment resistant patients mainly from the large urban areas. The London clinic is a national specialist service, and mostly sees cases of young people who have been through their local services, considered to be more severe and treatment-resistant, and who had waited many years for specialist treatment (Krebs et al., 2015). Australia utilizes both insurance-based and user-pays systems, as tax-funded healthcare is limited in accessibility due to strict entry criteria for mental health services and where tax-funded services are more often available in urban cities and centers, with limited or no service available in rural and regional areas. A large proportion of the families who seek treatment through the University clinic in Brisbane are families who “fall through the gap” if they fail to meet entry criteria for tax-funded services, do not live geographically close to services, or do not have the financial means to access insurance-based or user-pays systems. Altogether, these financial and organizational differences may well have affected participant retention. Overall, it is clear that the implementation of e-health solutions, such as BIP OCD, should be done with full consideration of the local healthcare idiosyncrasies.

The 3-month outcomes on both primary and secondary outcome measures were largely comparable to those of the previous BIP OCD trials, with similar within-group effect sizes and an observed additional improvement between post-treatment and the 3-month follow-up (Aspvall et al., 2018; Lenhard et al., 2017a; Lenhard et al., 2014). Although the validity of the results might have been affected by the number of participants attending the follow-up appointments or potential site differences, the estimated values are somewhat higher than the observed values and provide a more realistic estimate of the treatment effect. Further, the study was not conceived, or had statistical power, to examine site differences in treatment outcome, but visual inspection of the data indicated some potential site differences, as presented in the supplement. While this is an uncontrolled study, the results were encouraging and suggest that it may be possible to implement this treatment modality in regular healthcare settings with regular clinician-referred patients, even though further research is needed to establish the effectiveness of the treatment in these contexts. The optimal model for implementation of therapist-guided internet-based interventions is currently unclear and a matter of utmost importance, because it is unlikely that BIP OCD is suitable for all patients (Lenhard et al., 2018). One attractive model of healthcare delivery may be a stepped-care approach whereby patients with mild to moderate symptom severity who are willing to try this treatment format may be offered it in the first instance. In case of insufficient response, these patients could then be offered regular face-to-face CBT with a clinician, or more complex interventions in the context of a multidisciplinary team that can also offer additional treatment strategies, such as pharmacotherapy (Mataix-Cols and Marks, 2006). A stepped-care approach where counseling with bibliotherapy as the first treatment step was evaluated in a small trial of adults with OCD (Tolin et al., 2011), and our ongoing non-inferiority trial (Aspvall et al., 2019) is designed to directly address these important questions regarding ICBT for pediatric OCD.

The therapists were generally satisfied with the digital format of the treatment delivery and perceived to be a convenient way of receiving treatment for the families, with reduced travel distances, flexibility to work with the treatment when suitable, access treatment information on multiple occasions, and possibility to have a daily contact with the therapist. The BIP OCD treatment is also time efficient for the therapists with reduced therapist time and no late cancelations or missed appointments. Some therapists in this study experienced some difficulty in gauging exactly how much ERP the participants were actually completing. They also found it somewhat difficult to support participants using written text, as compared to traditional face-to-face format, as many young people did not write a lot of text. This is not so surprising as participants in ICBT tend to work more autonomously and written responses are sometimes limited in self-help treatments. Furthermore, many therapists thought that the BIP OCD treatment was harder to deliver to patients with more complex clinical symptoms such as mental compulsions, rigid rules, and reading/writing compulsions. Future development of BIP OCD may require attention to less common symptom presentations, which may be insufficiently covered in the current iteration of the intervention.

There were several limitations in this study that need to be acknowledged. First, this was an open study and it did not have a control group condition. Thus, some of the observed changes may not be entirely attributable to the intervention. Second, this study piloted the implementation of BIP OCD in three highly industrialized and wealthy cities; it cannot be automatically assumed that the results would be similar in other parts of the world, such as low or medium income countries (Demyttenaere et al., 2004), or countries with different healthcare systems (e.g. insurance based systems). Another limitation of this study was that slightly different inclusion- and exclusion criteria were used between the different sites, mainly due to practical reasons. This may have introduced some unwanted variability in the data. Further, the qualitative feedback was not formally analysed, since the information was not gathered in a structured way, and all therapists are co-authors of the study. Finally, there were no evaluation of clinician adherence in the current study, and patient adherence was pragmatically conceptualized as number of completed chapters and number of individuals classed as treatment completers. However, measuring adherence to e-health interventions may be more complex (Lenhard et al., 2019).

To conclude, the results in this study indicate that BIP OCD is a promising and potentially effective treatment for children and adolescents with OCD in a range of different healthcare settings in three different developed countries. This suggests that ICBT for pediatric OCD may be transferrable to outpatient clinics, though further evaluations are needed. Lessons learned are the need for continuous revision of the intervention, and the importance for future evaluations to gather data also for the non-completers. Additional improvements can be expected in BIP OCD from post-treatment to the 3-month follow-up and one opportunity for future trials is to test the treatment within a stepped care model. Implementation success is likely to be highly dependent on the specific healthcare context and on the adequate training of personnel delivering the treatment.

## Declaration of competing interest

Dr. Andersson reports receiving book royalties outside the submitted work. Dr. Turner reports receiving personal fees for editorial work from British Journal of Clinical Pharmacology and book royalties outside the submitted work. Prof Mataix-Cols reports receiving personal fees for editorial work from Elsevier and royalties for contributing articles to UpToDate, Inc., all outside the submitted work. The remaining authors report no conflicts of interest.

## Appendix A Supplementary data

S1. Observed mean and standard deviations by study site S2. Estimated baseline mean and change using multiple imputation by chained equations S3. Detailed feedback from the therapists, by study site. Image 1
