# Supplementary material for: Implementation of internet-delivered cognitive behaviour therapy for pediatric obsessive-compulsive disorder: Lessons from clinics in Sweden, United Kingdom and Australia
Source: Internet Interv. 2020 Jan 27;20:100308. doi: 10.1016/j.invent.2020.100308 (PMC7019117; doi:10.1016/j.invent.2020.100308)
Supplement: Supplementary file 1 — S1. Observed mean and standard deviations by study site S2. Estimated baseline mean and change using multiple imputation by chained equations S3. Detailed feedback from the therapists, by study site. [file mmc1.pdf]

S1. Observed means and standard deviations for the outcome measures, by study site

	Gothenburg						London						Brisbane					
	**Baseline**		**Post-treatment**		**3-month FU**		**Baseline**		**Post-treatment**		**3-month FU**		**Baseline**		**Post-treatment**		**3-month FU**	
**Measure**	**Mean (SD)**		**Mean (SD)**		**Mean (SD)**		**Mean (SD)**		**Mean (SD)**		**Mean (SD)**		**Mean (SD)**		**Mean (SD)**		**Mean (SD)**	
CY-BOCS	24.08	(3.23)	15.77	(6.57)	13.46	(9.99)	24.89	(4.73)	14.33	(7.89)	13.33	(7.61)	25.00	(2.87)	17.00	(7.62)	8.50	(0.71)
CGAS	48.77	(6.19)	59.15	(10.13)	63.54	(15.38)	52.33	(9.31)	61.80	(10.03)	62.83	(10.83)	57.56	(3.17)	66.75	(9.95)	79.50	(0.71)
ChOCI-R-P	31.54	(6.81)	18.92	(10.83)	14.50	(10.05)	20.86	(8.73)	20.40	(7.77)	19.80	(10.40)	21.11	(5.01)	15.75	(3.30)	9.00	-
WSAS-Y	15.31	(6.74)	8.67	(5.99)	6.50	(5.76)	16.00	(3.74)	13.25	(2.87)	12.25	(5.12)	9.11	(5.78)	6.00	(5.42)	4.00	-
WSAS-P	16.23	(8.07)	8.23	(7.66)	8.17	(7.57)	11.14	(9.49)	14.00	(12.03)	11.00	(9.54)	8.33	(8.41)	7.00	(2.45)	4.00	-

Abbreviations: CY-BOCS, Children Yale-Brown Obsessive-Compulsive Scale; CGAS, Children's Global Assessment Scale; ChOCI-R-P, Children's Obsessional Compulsive Inventory – Revised – Parent version; FU, follow-up; WSAS-Y, Work, and Social Adjustment Scale – Youth version, WSAS-P, Work, and Social Adjustment Scale – Parent version.

S2. Estimated baseline mean and change using multiple imputation by chained equations

		Baseline – post-treatment	Post-treatment – 3-month follow-up
**Measure**	**Estimated mean baseline**	**Estimated change**	**Estimated change**
CY-BOCS	24.58	15.83**	13.21*
CGAS	52.35	61.72**	65.17
ChOCI-R-P	25.52	18.16**	16.01
WSAS-Y	13.19	8.90**	7.73
WSAS-P	12.48	9.09	8.38

Abbreviations: CY-BOCS, Children Yale-Brown Obsessive Compulsive Scale; CGAS, Children's Global Assessment Scale; ChOCI-R-P, Children's Obsessional Compulsive Inventory – Revised – Parent version; WSAS-Y, Work, and Social Adjustment Scale – Youth version, WSAS-P, Work, and Social Adjustment Scale – Parent version.
*$P < .01$, **$P < .001$

S3. Detailed feedback from the therapists, by study site.

Question	Gothenburg	London	Brisbane
**What are the advantages of BIP OCD?**	Pedagogical and appealing programme	Requires less therapist input	BIP offers advantages of giving families choice of treatment delivery
	Convenient for both patients and therapists who can work with the programme when they have time	Convenience – no travel required for families and they can complete sessions at any time they want	Convenience of not needing to travel to appointments and therefore reduced time
	Time efficient for the therapist while having the possibility to daily contact with the patients	Encourages young people to take ownership over their recovery	Families able to access the session information on multiple occasions
	Help the therapist to keep a clear structure for the treatment	In a format that young people find accessible	Technology is more appealing for many children and young people than workbooks
	Accessible – lower threshold to receiving care	Good for young people who struggle to speak in sessions e.g. socially anxious	The programme allows for parents and their children to work together if they wanted to i.e. both reading the information together and learning together
	Parents are highly involved and have the main responsibility for the treatment		Parents wanted information and many welcomed the parent chapters
	Parents are strengthened in their parental role		It made accessing mental health services largely stigma free for many young people
	Easier for the parents to use the knowledge after treatment completion (more similar to the ”real world”)		Information was excellent and translation was very well written
	Many modalities (a lot of visual information), and that the information is accessible after treatment completion		Lots of scope for back-up support by therapist if the family chose to use it, however many didn’t choose to use it

	The therapists don'thave any downtime, due to cancellations or missed appointments		Internet is accessible for many young people in western societies
	Suitable for patients with attention difficulties		The use of case studies and video's are engaging and helpful
	The therapist have more time to reflect and can ask for peer-support before answering the patients		
**What are the disadvantages of BIP OCD?**	Less control and influence as therapist	Possibly not so good for young people who are ambivalent about treatment – they may be likely to drop out.	Parent chapters were more limited than child chapters and parents very keen for lots of advice, information, and ideas about how they could help
	Some patients would need more support with exposures	As a therapist it can be hard to gauge exactly what the young person is doing and how well they are progressing	Offering internet treatment made many parents think that their child would do it, when actually many children were half-hearted about it and I think often agreed simply to please their parents
	No possibility for in vivo therapist-guided ERP	Can be trickier with young people who have less classical OCD symptoms and/or significant comorbid problems as the materials are not geared towards them – in these cases the therapist may have to spend longer communicating with the young person	It is very difficult for young people with very complex symptoms with many rules and/or covert neutralizing strategies to fully understand and appreciate what they needed to do in order to be successful
	More difficult for patients with more complex symptoms	Generally, I found engagement to be difficult. May reflect the kind of cases (i.e. treatment-resistant cases who had had CBT before and were ambivalent about further CBT)	Young people often wrote very limited information in their response boxes, so it was difficult to engage them "virtually"

	Some patients don't manage to take responsibility for their own treatment	Difficult to monitor ongoing risk of suicidality. It might be helpful to have the option to include weekly risk questionnaire, or perhaps anyone with a history of suicidality should not be offered iCBT.	Some of the graphics may be a bit simple for older teenagers and may be perceived as babyish
	Uneven therapist time, difficult to know how much time is needed (face-to-face CBT sessions are have a more clear structure)		Sometimes difficult for young people to conceptualize their symptoms as being associated with different disorders (e.g. thinking tics are OCD symptoms) when they create their problem lists
	Difficult for patients with OCD-symptoms regarding computers and reading/writing to participate		Hard to tell if young people are fully engaged with the online content or whether they may be listening to music or messaging friends at the same time as completing chapters – high potential for divided attention from young people
	Some patients or parents had a strong wish to see a therapist		Access to fast speed internet is still very limited in some rural and regional areas meaning the programme doesn't run as effectively as it could.
	Limited to patients and parents who has sufficient knowledge in Swedish		The authors of the programme have advised that the cost of programme development and maintenance means it needs large amounts of dollars to keep it running – likely to need partnership with IT as well as government bodies. How to fully engage with long-term partners for ongoing funding is always difficult. Would be sad

			to lose the English language version.
**What concerns do you have regarding BIP OCD?**		Difficult to monitor ongoing risk of suicidality. It might be helpful to have the option to include weekly risk questionnaire, or perhaps anyone with a history of suicidality should not be offered iCBT.	The authors of the programme have advised that the cost of programme development and maintenance means it needs large amounts of dollars to keep it running – likely to need partnership with IT as well as government bodies. How to fully engage with long-term partners for ongoing funding is always difficult. Would be sad to lose the English language version.
**What are you satisfied with?**		Clear and easy to follow. Young people liked the videos. Very few technical problems	Really liked the program.
**If you would continue with BIP OCD in the clinic, what do you consider is important in implementing the treatment?**	Education in the technical aspects of BIP and the therapist role is needed, for example what is the optimal level for the therapist-support	Having guidelines regarding who should and shouldn't be offered CBT (I'm not sure if it is suitable for those who have failed to respond to good quality, face-to-face CBT for example)	Better and more careful screening of participant suitability and ensuring that both parents and young people are fully cognizant of the time commitments and time needs for being able to complete the programme successfully
	Therapists should have knowledge about CBT/ERP and OCD to be an internet-therapist	In terms of content, there are a few materials I would like to change / add. I think it would be helpful to structure exposure task around a hierarchy – this would help with tracking progress as well as guiding the exposure. I also think it would be helpful to have an optional worksheet and video on "normalizing intrusive thoughts", particular aimed at young	Giving families the option of telephone or online support if they require it, and potentially being able to add in video support for "live" exposure when symptoms are very complex as young people can get stuck and it can be very helpful for therapist to ask lots of questions around certain symptoms [just using an online forum often means young people will give only very short or simple

		people with “taboo” obsessions.	responses which may not capture the complexity of certain symptoms]
	Important to reflect on which patients who should be offered ICBT	More sessions on the anxiety rating scale and devising that	Text alerts to remind young people and their parents to log in and work on the chapters
	Reflect on organizational aspects if implementing ICBT		Families uncertain of how to manage comorbid disorders alongside the program, so potentially better advice on putting “other issues” on hold or a plan for managing other issues that arise [e.g. symptoms at school].
